# Phenotypic and Genotypic Characterization of AmpC Beta-Lactamase in Clinical Isolates of Pseudomonas aeruginosa Findings From a Tertiary Care Hospital

**DOI:** 10.7759/cureus.65185

**Published:** 2024-07-23

**Authors:** Suvarna A Yadav, Satyajeet K Pawar, Kailas D Datkhile, Shivaji T Mohite, Satish R Patil, Ashwini L More

**Affiliations:** 1 Microbiology, Krishna Institute of Medical Sciences, Krishna Vishwa Vidhyapeeth (Deemed to be University), Karad, IND; 2 Microbiology, Krishna Institute of Medical Science, Krishna Vishwa Vidyapeeth (Deemed to be University), Karad, IND; 3 Molecular Biology and Genetics, Krishna Institute of Allied Sciences, Krishna Vishwa Vidyapeeth (Deemed to be University), Karad, IND; 4 Microbiology, Krishna Institute of Medical Sciences, Krishna Vishwa Vidyapeeth (Deemed to be University), Karad, IND

**Keywords:** blacmy gene, blapdc gene, ampc β-lactamase, antibiotic resistance, pseudomonas aeruginosa

## Abstract

Background and aim

*Pseudomonas aeruginosa* is an opportunistic pathogen responsible for various healthcare-related infections, which are difficult to treat due to intrinsic and acquired resistance. This study aimed to investigate AmpC β-lactamase production using phenotypic and genotypic methods in *Pseudomonas aeruginosa* strains isolated from a tertiary care hospital in Karad, Maharashtra, India.

Material and methods

Over one year, a descriptive cross-sectional study was conducted at the Department of Microbiology, Krishna Institute Medical Sciences, Krishna Vishwa Vidyapeeth, Karad. Phenotypic detection of AmpC beta-lactamase was performed using the Cefoxitin-Cloxacillin Double-Disc Synergy Test method, and genotypic detection was conducted using conventional polymerase chain reaction (PCR) targeting the bla *Pseudomonas*-derived cephalosporinases (PDC) and bla cephamycinase (CMY) genes.

Results

Out of 205 clinical isolates of *Pseudomonas aeruginosa*, 110 (53.66%) showed AmpC production phenotypically, while 86 (41.95%) were positive genotypically. The blaPDC gene was detected in 36.10% of isolates, and the blaCMY gene in 10.73% of isolates.

Conclusions

The study findings indicate that AmpC-β-lactamase stands out as the primary resistance mechanism in strains of *Pseudomonas aeruginosa* isolated from the hospital. PCR study concluded that blaPDC (36.10 %) was the leading gene responsible for AmpC synthesis among study isolates. Early detection of AmpC beta-lactamase production by employing phenotypic and genotypic methods is crucial for detecting antibiotic resistance. This dual approach enables healthcare professionals to decide on the most effective antibiotics and mitigate the development of resistance.

## Introduction

*Pseudomonas aeruginosa* is an opportunistic pathogen responsible for various healthcare-related infections [1]. These infections are challenging to treat due to the bacteria's intrinsic and acquired resistance mechanisms [2]. The improper and unnecessary use of β-lactam antibiotics exacerbates bacterial resistance, leading to the selection of extended-spectrum β-lactamases (ESBL), AmpC β-lactamases, and metallo-β-lactamases (MBL), which pose significant challenges in antimicrobial therapy [3]. *Pseudomonas aeruginosa*, typically susceptible to ceftazidime, aztreonam, and carboxypenicillins, can develop resistance to third-generation cephalosporins through the hyperproduction of AmpC beta-lactamase [2]. AmpC β-lactamases hydrolyze cephalosporin antibiotics and are resistant to clavulanic acid. Unlike ESBLs, AmpC β-lactamases can deactivate a range of antimicrobials, including monobactams, aminopenicillins, cephamycins, and cephalosporins [4]. Both chromosomal and plasmid-mediated genes can produce these enzymes [5].

AmpC β-lactamases are class C enzymes according to the Ambler classification, they have serine residues that facilitate catalysis in their active site [6]. There are three types of resistance mechanisms by AmpC β-lactamases can be distinguished: i) inducible resistance by chromosomally encoded AmpC genes, accountable for resistance in bacteria like *Serratia marcescens, Enterobacter cloacae, Citrobacter freundii, Pseudomonas aeruginosa,* etc. ii) non-inducible chromosomal resistance caused by mutations in the promoter and attenuator mechanisms (e.g., *Acinetobacter baumannii, Shigella *species*, and Escherichia coli*), iii) or resistance mediated by plasmids (e.g., *Salmonella *species*, Klebsiella pneumonia, E. coli,* etc.) [7].

Even infections produced by originally susceptible isolates can develop considerable AmpC synthesis and β-lactam resistance as a result of exposure to β-lactams. Treatment considerations are complicated by species and β-lactam variations in the possibility of generating AmpC formation [8]. Three proteins, AmpG, AmpD, and AmpR, are involved in the regulation of AmpC β-lactamase induction. Mutations in the structural AmpD genes result in hyper-inducibility and overproduction of the AmpC enzyme [9]. The main resistance against β-lactam drugs in *Pseudomonas aeruginosa* involves mutations boosting chromosomal AmpC production, and selecting resistant mutants. *Pseudomonas aeruginosa *also produces chromosomal cephalosporinases (​​​​​[*Pseudomonas*-derived cephalosporinases (PDC)] with extended-spectrum activity. Plasmid-mediated AmpC emergence adds to the challenge of treating *Pseudomonas aeruginosa* infections [10].

Plasmid-mediated class C enzymes are named according to various criteria. Some names for them include Ambler class C (ACC) or AmpC type (ACT), which are β-lactamase types; cefoxitin (FOX), or cephamycins (CMY), which are antibiotics they resist; and Dhahran Hospital (DHA) or Miriam Hospital (MIR-1), which are the names of the hospitals where they were found. In rare cases, they are even given patient names, like Bilal (BIL-1) [11]. South Korea (CMY-1) is where the first pAmpC was found in 1989. Many other pAmpCs (FOX, CIT, MOX, DHA, EBC, and ACC) have since been characterized; the most frequent subtype is the CMY-2 enzyme (CIT-type) [12].

The study investigated beta-lactamase-mediated resistance in hospital isolates of *Pseudomonas aeruginosa*. "The primary objective of this study is to investigate the prevalence and distribution of AmpC beta-lactamase-producing *Pseudomonas aeruginosa* in a tertiary care hospital setting in Karad, Maharashtra, India." "The secondary objectives include phenotypic and genotypic characterization of AmpC beta-lactamase production and identification of the specific genes (blaPDC and blaCMY) responsible for this resistance.

The prevalence of multidrug-resistant (MDR) infections is rapidly increasing in hospital settings, largely attributed to the extensive utilization of broad-spectrum cephalosporins, which hampers the efficacy of control measures. In this regard, this study aimed to explore the production of AmpC β-lactamase in the strains of *Pseudomonas aeruginosa *isolated from a tertiary care hospital in Karad, Maharashtra, India.

## Materials and methods

Study design

This study was designed as a cross-sectional analysis to investigate the prevalence and distribution of AmpC genes in *Pseudomonas aeruginosa* isolates.

Study period

The research was conducted over 18 months, from September 2021 to February 2023.

Sample size

Two hundred and five *Pseudomonas aeruginosa* isolates were collected and analyzed during the study period.

Data collection

Clinical specimens from Krishna Hospital and Medical Research Center (KH & MRC) were collected and processed at the Department of Microbiology, Krishna Institute Medical Sciences, Karad.

Variables

The primary variables in this study included the presence of AmpC genes (e.g., blaPDC, and blaCMY), antibiotic susceptibility profiles, and patient demographics such as age, gender, etc.

Inclusion criteria

Non-repetitive clinical isolates of *Pseudomonas aeruginosa* isolated in the study period were included.

These clinical specimens were processed as per standard guidelines, the specimens were inoculated on blood agar and chocolate agar, followed by MacConkey agar. The plates were incubated at 37°C for 24 hours, and colony morphology was evaluated [13]. Non-lactose fermenting (NLF) colonies from MacConkey agar were identified using oxidase testing. A total of 205 isolates of *Pseudomonas aeruginosa* were obtained. Antimicrobial susceptibility testing (AST) and identification were done using the VITEK-2 COMPACT system, with interpretations based on Clinical and Laboratory Standards Institute (CLSI) guidelines [14]. For quality control, *Pseudomonas aeruginosa* American Type Culture Collection (ATCC) 27853 was used. Multi-drug resistance was identified as resistance to more than three antimicrobial classes.

Phenotypic characterization

Phenotypic detection of AmpC β-lactamase production in selected isolates was done using the cefoxitin-cloxacillin double-disc synergy test (CC-DDS). This test is based on the inhibitory effect of cloxacillin on AmpC production. A suspension of isolates equivalent to a 0.5 McFarland turbidity standard was inoculated onto the Mueller Hinton agar plate. In that, one disc of cefoxitin (30μg) and another disc containing cefoxitin-cloxacillin (30μg/200μg), (Hi-Media Laboratories Pvt. Limited, Mumbai, India) were placed at a distance of 20 mm between their centers followed by incubation at 37 °C for 24 h. A difference of at least 4 mm in inhibition zone diameter around cefoxitin-cloxacillin discs compared to the zone around cefoxitin discs was considered as an AmpC-producing strain [15,16]. This method is suitable for detecting inducible (plasmid-mediated) AmpC beta-lactamase.

Genotypic characterization

The AmpC-producing isolates were analyzed for the presence of AmpC genes using polymerase chain reactions targeting blaPDC gene and blaCMY gene. Each isolate was cultured on nutrient agar and incubated at 37°C for 18-24 hr. Colonies were suspended in 10 ml nutrient broth and incubated overnight with shaking (200 rpm) at 37°C. Then the cells were harvested by concentration at 13000 rpm. The total DNA was extracted using the HiPurA Bacterial Genomic DNA Miniprep Kit from Himedia, Mumbai, from the pure culture of clinical isolates of *Pseudomonas aeruginosa*. Chromosomal DNA was extracted according to the manufacturer's instructions and extracted DNA was assessed for the concentration and purity and kept at -20°C.

For detecting the blaPDC gene and blaCMY gene, specific primers for the genes were amplified with the extracted bacterial DNA as the template. Amplification was performed in Eppendorf Mastercycler (Eppendorf, Germany), in a 20 μl reaction using the thermal programme and specific primer details described in Table 1. Reaction mixture containing 1 μl of DNA template, with a master mix (19 μl) which contains 1 U of Taq polymerase enzyme (0.5 μl), dNTPs (0.5 μl), Taq buffer (2 μl), Molecular biology grade water (15 μl), and 0.5 μl of each forward primer and reverse primer (20 pmol), Amplified PCR products were separated by electrophoresis on a 1.5% agarose gel stained with ethidium bromide at 100V for 45 min. A 100bp DNA ladder (BR Biochem) was used as a size marker and the addition of water instead of a DNA template served as the negative control. The gel images were taken under ultraviolet light using a gel documentation system (BIO-RAD, USA). The expected bands were determined and documented.

**Table 1:** Details of the primers and thermal program used for the amplification of genes PDC: Pseudomonas-derived cephalosporinases, CMY: cephamycinase.

Sr. No.	Genes	Primer sequence (5'-3')	Product Size	Ref.	Reaction conditions		
					PCR Steps	Temp. & Time	Cycles
1	blaPDC	F: AGAAGGACCAGGCACAGATC	671bp	[17]	Initial denaturation	95°C- 5mins	
		R: CTCGGCATTGGGATAGTTGC			Denaturation	95°C- 30sec	
					Annealing	60°C- 1min	35
					Extension	72°C- 1min	
					Final Extension	72°C- 10mins	
					Holding	4°C-∞	
2	blaCMY	F: CTGCACTTAGCCACCTATAC	650bp	[17]	Initial denaturation	95°C- 10mins	
		R: CCGTTTTATGCACCCATGAG			Denaturation	95°C- 1min	
					Annealing	53°C- 1min	35
					Extension	72°C- 1min	
					Final Extension	72°C- 10mins	
					Holding	4°C-∞	

Sequencing of PCR products 

To determine the full-length sequence purified PCR products were subjected to sequence determination using respective amplification primers, according to the above protocol. Amplified PCR products of Gene blaPDC and blaCMY were purified by using PureLinkTM Quick Gel Extraction and PCR Purification Combo Kit (Thermo Fisher) and sequencing was done, using the Sanger dideoxynucleotide sequencing method. Sequences were analyzed for homology by using the National Center for Biotechnology Information GenBank database. Basic local alignment search tool (BLAST) analysis was performed to search for homologous sequences in the GenBank database, and nucleotide sequences were submitted using Bank It submission tool to obtain accession numbers.

Statistical analysis

After entering the data into an Excel sheet and analyzed using Microsoft Excel, the data was expressed in percentages, and tables were prepared for different objectives.

## Results

In this study, 205 consecutive, non-repetitive *Pseudomonas aeruginosa *clinical isolates were obtained from various clinical specimens such as pus (n=90), urine (n=55), endotracheal secretion (n=30), sputum (n=15), body fluid (n=5), catheter tip (n=4), blood (n=3), and tracheostomy tube (n=3). AmpC production was seen in 114(55.61%) isolates by phenotypic method, and a Genotype study for AmpC production showed positive results in 86(41.95%) isolates. (Table 2)

**Table 2:** AmpC production among Pseudomonas aeruginosa isolates

Phenotype (%)	Genotype (%)	
	Negative	Positive
Negative 91(44.39)	60(29.27)	31(15.12)
Positive 114(55.61)	59(28.78)	55(26.83)
Total 205	119(58.05)	86(41.95)

Among 86 AmpC-positive isolates 73 (84.88%) were from inpatient departments and 13 (15.12%) were from outpatient departments. Antibiotic susceptibility pattern of these AmpC-positive isolates of *Pseudomonas aeruginosa* showed high resistance towards antipseudomonal penicillins followed by beta-lactamase inhibitors such as ticarcillin + clavulanic acid 77(89.53%) and piperacillin + tazobactam 52(60.47%). Resistance towards carbapenem was doripenem 55(63.95%) and meropenem 45(52.33%) and 4th generation cephalosporin i.e. cefepime 45(52.33%).

Amplification of AmpC genes (blaPDC and blaCMY)

**Figure 1:**
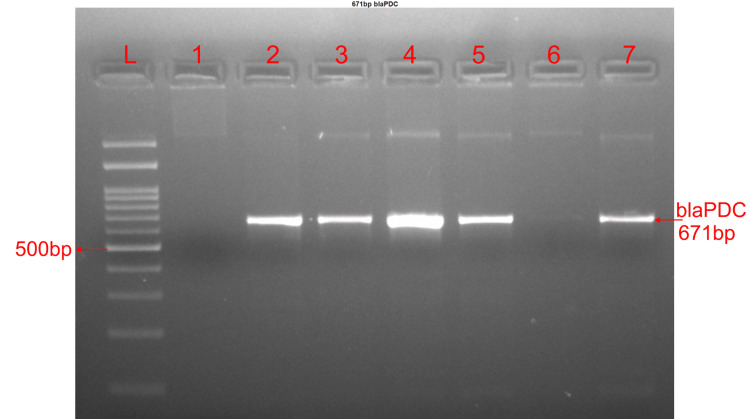
Agarose gel image of blaPDC gene having band size 671bp Lane L was loaded with the 100bp DNA ladder, and Lane 1 with water as a negative control.  Lanes 2,3,4,5 and 7 had positive results, and lane 6 had negative results.

**Figure 2:**
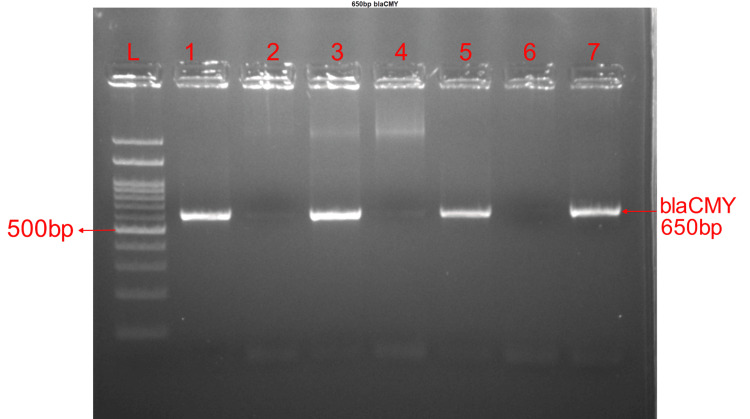
Agarose gel image of blaCMY gene having band size 650bp Lane L was loaded with the 100bp DNA ladder, lanes 1,3,5 and 7 had positive results, and lanes 2,4, and 6 had negative results.

The expected band size of 671bp was observed on agarose gel electrophoresis of amplified products of blaPDC gene amplicons (Figure 1). The amplification was detected in 74/205 (36.10%) isolates for blaPDC and the amplification of blaCMY gene was detected in 22/205 (10.73%) isolates, (Table 3) expected band size of 650bp was observed on agarose gel electrophoresis of the blaCMY gene amplicons (Figure 2).

**Table 3:** Distribution of AmpC genes in Pseudomonas aeruginosa (n=205) PDC: Pseudomonas-derived cephalosporinases, CMY: cephamycinase.

AmpC Genes	Number	Percentage
blaPDC	64	31.22
blaCMY	12	5.85
blaPDC+blaCMY	10	4.88
Total	86	41.95

Distribution of AmpC genes among 205 study isolates of *Pseudomonas aeruginosa *showed that only blaPDC amplification was detected in 64 (31.22%) isolates and blaCMY gene amplification was detected in 12 (5.85%) isolates and amplification of both genes was detected in 10 (4.88%) isolates.

Blast analysis & accession numbers

Genes sequenced in this study were deposited in the National Center for Biotechnology Information (NCBI) and were assigned with different accession numbers: PP410441 (SY114) for blaPDC and PP410442 (SY206) blaCMY, and search for BLAST gave 99% similarity to homologous sequences in the GenBank database.

## Discussion

Resistance to carbapenems, cephalosporins, cephamycins, and monobactams is caused by overproduction of AmpC. Mutations in regulatory genes regulating AmpC expression or transient transcription activation in response to β-lactam exposure may cause this overexpression. Although isolates that overexpress AmpC are still vulnerable to cefepime and cefpirome, they frequently show resistance against traditional β-lactamase inhibitors [18]. AmpC producers are important in treatment decisions, but detecting them lacks standardized guidelines. Due to challenges in phenotypic detection, AmpC beta-lactamases are mostly unknown, as per scientist Inamdar et al. CC-DDS approach against other phenotypic confirmatory methods, showed a higher detection rate and was simple to use [16]. Our study reported a slightly low incidence i.e. 55.61% compared to Upadhyay et al. discovered that AmpC production was present in 59.4% of isolates of *Pseudomonas aeruginosa* from Varanasi [4]. The incidence of AmpC production by Agarwal et al. was 46.00% in Kanpur, Uttar Pradesh [19]. 

AmpC β-lactamases mediated by plasmids present a significant obstacle to infection control since the AmpC gene is highly transmissible to other bacterial species and can express itself at higher levels [15]. In this study, we observed among AmpC-positive isolates 74 (84.88%) were from the inpatient department. Similar results were observed by Madhumati et al. 84 (82%) were from inpatients. A significant percentage of isolates harboring AmpC was found in isolates collected from inpatients, confirming the nosocomial significance of this pathogen [20]. People around the world can easily get nosocomial infections due to poor hygiene. In hospitals, the most serious pseudomonal infections occur, either on the hands of healthcare workers or through contaminated and improperly cleaned equipment.

Our study isolated both AmpC-type variants, namely blaPDC and plasmid-mediated cephamycins (CMY), from chromosomal-extracted DNA. This suggests that plasmid-mediated AmpC enzymes originate from chromosomal enzymes. These enzymes exhibit similar traits and resistance to their chromosome-derived counterparts. Certain cephalosporins can induce AmpC expression, leading to resistance. The resistance-causing genes can transfer between the chromosome and plasmid through mechanisms like insertion sequences, integrons, and transposons. Consequently, plasmid-mediated AmpC resistance may pose greater risks compared to chromosome-mediated AmpC resistance [21]. In the present study, 64 out of 205 isolates (31.22%) have the blaPDC gene, 12 isolates (5.85%) have the blaCMY gene, and both together genes in 10 isolates (4.88%). A similar study by Bharti et al. reported from Solan, Himachal Pradesh, India, 21.15% of blaPDC gene amplification in *Pseudomonas aeruginosa* but they did not achieve amplification of blaCMY [17]. Another study by Ignti et al. from Silchar, Assam, India reported that 23% of isolates showed blaPDC gene [22]. Overall, the significance of this study is the potential to advance clinical infection care, better understand *Pseudomonas aeruginosa* antibiotic resistance, and educate public health initiatives to counteract the escalating problem of antibiotic resistance. 

Detection of AmpC beta-lactamase in *Pseudomonas aeruginosa* by phenotypic and genotypic methods has its strengths and limitations. Both methods may provide a more comprehensive understanding of AmpC production and resistance patterns. Together, they can accurately detect both gene presence and expression, offering a more complete picture of bacterial resistance.

Limitations

Genotypic characterization in the study was limited to only two genes. Other genes such as novel or less common genetic variants of AmpC beta-lactamases were not included. *Pseudomonas aeruginosa* has multiple resistance mechanisms, this study focused only on AmpC beta-lactamases, other resistance mechanisms, such as efflux pumps or porin mutations, were not in the scope of the study.

## Conclusions

The study concluded that AmpC-β-lactamase is the primary resistance mechanism in *Pseudomonas aeruginosa* strains isolated from a hospital setting. A significant proportion of these clinical isolates were identified as AmpC producers using both phenotypic and genotypic methods. This dual approach allows healthcare professionals to choose the most effective antibiotics and mitigate resistance development. The study emphasizes the necessity of continuous surveillance and infection control measures to prevent the spread of resistant strains in hospitals. Resistance patterns indicated that AmpC production is linked to higher resistance against broad-spectrum antibiotics, complicating treatment options. The prevalence of specific genotype patterns suggests the evolution or spread of genetic elements within bacterial populations, highlighting the need for accurate detection methods to enhance clinical management and antibiotic stewardship. AmpC beta-lactamase detection using both phenotypic and genotypic methods is crucial in clinical microbiology for guiding effective treatment, preventing the spread of resistant infections, and contributing to the broader understanding of antimicrobial resistance mechanisms.

## References

[1] Lister PD, Wolter DJ, Hanson ND (2009). Antibacterial-resistant Pseudomonas aeruginosa: clinical impact and complex regulation of chromosomally encoded resistance mechanisms. Clin Microbiol Rev.

[2] Strateva T, Yordanov D (2009). Pseudomonas aeruginosa - a phenomenon of bacterial resistance. J Med Microbiol.

[3] Shivanna V, Rao A (2017). Detection of co-existence of β-lactamases in Gram negative bacteria using disc potentiation tests. Indian J Microbiol Res.

[4] Upadhyay S, Sen MR, Bhattacharjee A (2010). Presence of different beta-lactamase classes among clinical isolates of Pseudomonas aeruginosa expressing AmpC beta-lactamase enzyme. J Infect Dev Ctries.

[5] Chanu TR, Shah PK, Soni S, Ghosh AN (2023). Phenotypic detection of extended spectrum, AmpC, metallo beta-lactamases and their coexistence in clinical isolates of commonly isolated gram negativebacteria in GKGH hospital, Bhuj. Int J Med Microbiol Trop Dis.

[6] Bush K, Jacoby GA (2010). Updated functional classification of beta-lactamases. Antimicrob Agents Chemother.

[7] Jacoby GA (2009). AmpC beta-lactamases. Clin Microbiol Rev.

[8] Tamma PD, Doi Y, Bonomo RA, Johnson JK, Simner PJ (2019). A primer on AmpC β-lactamases: necessary knowledge for an increasingly multidrug-resistant world. Clin Infect Dis.

[9] Schmidtke AJ, Hanson ND (2006). Model system to evaluate the effect of ampD mutations on AmpC-mediated beta-lactam resistance. Antimicrob Agents Chemother.

[10] Upadhyay S, Mishra S, Sen MR, Banerjee T, Bhattacharjee A (2013). Co-existence of Pseudomonas-derived cephalosporinase among plasmid encoded CMY-2 harbouring isolates of Pseudomonas aeruginosa in north India. Indian J Med Microbiol.

[11] Philippon A, Arlet G, Jacoby GA (2002). Plasmid-determined AmpC-type beta-lactamases. Antimicrob Agents Chemother.

[12] Oliveira C, Amador P, Prudêncio C, Tomaz CT, Tavares-Ratado P, Fernandes R (2019). ESBL and AmpC β-lactamases in clinical strains of Escherichia coli from Serra da Estrela, Portugal. Medicina (Kaunas).

[13] Pawar SK, Mohite ST, Datkhile KD, Patil MN, Kakade SV (2020). Rising Threat of OXA-48 and other carbapenemase encoding genes among carbapenem resistant Enterobacteriaceae in India. J Pure Appl Microbiol.

[14] CLSI CLSI (2024). CLSI M100 Performance Standards for Antimicrobial Susceptibility Testing, 34th edition. Clinical and Laboratory Standards Institute.

[15] Mohd Khari FI, Karunakaran R, Rosli R, Tee Tay S (2016). Genotypic and phenotypic detection of AmpC β-lactamases in Enterobacter spp. isolated from a teaching hospital in Malaysia. PLoS One.

[16] Inamdar DP, Anuradha B (2020). Phenotypic methods for detection of Amp C β lactamases in gram-negative clinical isolates of a tertiary care hospital. Indian J Microbiol Res.

[17] Bharti Bharti, Minhas N and Sharma PC (2016). Molecular characterization of pseudomonas aeruginosa isolates recovered from human patients in Himachal Pradesh (India) for selective genes: extended spectrum β-lactamase (ESBL), ampicillin class c (AMPC) and metallo β-lactamase (MBL) genes. Int J Pharm Sci Res.

[18] Mizrahi A, Delerue T, Morel H, Le Monnier A, Carbonnelle E, Pilmis B, Zahar JR (2020). Infections caused by naturally AmpC-producing Enterobacteriaceae: can we use third-generation cephalosporins? A narrative review. Int J Antimicrob Agents.

[19] Agrawal A, Srivastava N, Kumar D, Rashmi Rashmi, Bhati N (2017). AmpC β-lactamase production in Pseudomonas aeruginosa: a threat. Int J Curr Microbiol Appl Sci.

[20] Madhumati B, Leela Rani LR, Ranjini CY, Rajendran R (2015). Prevalence of AmpC beta lactamases among gram negative bacterial isolates in a tertiary care hospital. Int J Curr Microbiol App Sci.

[21] Ronni Mol P, Shanthi G, Al-Mahmeed A, Bindayna KM, Shahid M (2022). Class C type β-lactamases (AmpC β-lactamases). In beta-lactam resistance in gram-negative bacteria: threats and challenges.

[22] Ingti B, Krishnatreya DB, Maurya AP, Dhar Chanda D, Chakravarty A, Bhattacharjee A (2017). Role of inducers in detection of bla(PDC)-mediated oxyimino-cephalosporin resistance in Pseudomonas aeruginosa. Indian J Med Res.

